# Anti-Neuroinflammatory Effects and Mechanism of Action of *Fructus ligustri lucidi* Extract in BV2 Microglia

**DOI:** 10.3390/plants10040688

**Published:** 2021-04-02

**Authors:** Yeon Ju Kim, Sung Yun Park, Young Jun Koh, Ju-Hee Lee

**Affiliations:** 1 Department of Medical Biotechnology, Dongguk University, Seoul 04620, Korea; a_ajoo@naver.com; 2 College of Korean Medicine, Dongguk University, Goyang 10326, Korea; bmepark@dongguk.ac.kr; 3 GI Innovation, Inc., Seoul 05855, Korea

**Keywords:** *Fructus ligustri lucidi*, microglia, neuroinflammation, polarization, HO-1

## Abstract

For centuries, *Fructus ligustri lucidi* (FLL; the fruit of *Ligustrum lucidum* Aiton or *Ligustrum japonicum* Thunb.) has been commonly used in traditional Chinese medicine for treating hepatitis and aging-related symptoms and in traditional Korean medicine to detoxify kidneys and the liver. Pharmacological research has shown FLL has antioxidant, anti-inflammatory, anticancer, anti-osteoporosis, and hepatoprotective activities. This study was undertaken to investigate the effects of FLL extract (FLLE) on neuroinflammation. After setting a non-toxic concentration using MTT [3-(4,5-dimethylthiazol-2-yl)-2,5-diphenyl-tetrazolium bromide] assay data, we investigated the effects of FLLE using Western blotting, cell migration, enzyme-linked immunosorbent assay, a nitric oxide (NO) assay, and immunofluorescence staining in lipopolysaccharide (LPS)-stimulated murine BV2 microglial cells. FLLE was non-toxic to BV2 cells up to a concentration of 500 μg/mL and concentration-dependently inhibited the production of NO and prostaglandin E_2_ and the protein levels of inducible nitric oxide synthase and cyclooxygenase-2 under LPS-induced inflammatory conditions. It also inhibited the secretion of the inflammatory cytokines tumor necrosis factor-α (TNF-α) and interleukin-6 (IL-6). Furthermore, FLLE pretreatment attenuated LPS-induced increases of CD68 (a marker of microglia activation) and suppressed the activation of mitogen-activated protein kinases (MAPKs) and nuclear factor-kappa B (NF-κB) signaling pathways in LPS-stimulated BV2 cells, and significantly increased heme oxygenase (HO)-1 levels. FLLE also reduced the LPS-induced increase in the migratory ability of BV2 cells and the phosphorylation of vascular endothelial growth factor receptor 1. Collectively, FLLE effectively inhibited inflammatory response by suppressing the MAPK and NF-κB signaling pathways and inducing HO-1 in LPS-stimulated BV2 microglial cells. Our findings provide a scientific basis for further study of FLL as a candidate for preventing or alleviating neuroinflammation.

## 1. Introduction

In concert with an aging population, the number of people suffering from various brain diseases such as Alzheimer’s disease, Parkinson’s disease, and multiple sclerosis continues to increase and this presents an enormous socioeconomic challenge for the management and treatment of brain disease [1,2,3]. Despite the numerous studies conducted to date, the pathogenesis of brain diseases has not been fully elucidated, and no treatment has been developed.

Neuroinflammation is an immune response that protects brain cells against harmful stimuli, such as infections, stresses, injuries, and aging, and is one of the most common symptoms or hallmarks in neurodegenerative diseases including Alzheimer’s, Parkinson’s, and Huntington’s diseases and multiple sclerosis [4]. Accumulating evidence supports the view that uncontrolled chronic neuroinflammation is linked to the progression of neurodegenerative diseases [5]. Glial cells include astrocytes, microglia, oligodendrocytes, and neuron glia antigen-2 glia and play distinct roles during neuroinflammation [4,6]. Of these, microglia are brain-specific macrophages residing in the central nervous system (CNS) and act as the predominant modulators of neuroinflammation [7]. Microglia also play a pivotal role in the maintenance of homeostasis in the CNS by recognizing and removing exogenous pathogens or damaged cells and by secreting nerve growth factors [8,9]. Activation of microglia by pathogens such as amyloid-associated proteins or lipopolysaccharide (LPS, a bacterial endotoxin) induces neuroinflammatory response by promoting the secretion of inflammatory mediators (e.g., nitric oxide (NO) and prostaglandin E_2_ (PGE_2_)), proinflammatory cytokines, and reactive oxygen species [10]. Excessive neuroinflammatory response causes neurotoxicity, induces neuronal damage, and eventually leads to neuronal degeneration [11,12]. Thus, the suppression of microglial overactivation is viewed as an important target for the prevention and treatment of neurodegenerative diseases. Unfortunately, drugs that are currently used to treat degenerative brain diseases have toxic and other side effects and, thus, studies are being conducted to control the inflammatory response using relatively safe natural products.

*Fructus ligustri lucidi* (FLL, known in Chinese as Nu Zhen Zi or Nvzhenzi) is the dried ripe fruit of *Ligustrum lucidum* Aiton or *Ligustrum japonicum* Thunb., which are both members of the Oleaceae family [13,14]. FLL has been often prescribed in doses of 6–15 g per day to treat several diseases in traditional Korean medicine [15]. Traditionally, it is considered to counteract the effects of preventing aging by tonifying the liver and kidneys, improving vision, and preventing hair graying [14,16]. Er Zhi Wan is a representative prescription and contains FLL and the herb *Ecliptae Herba* (*Eclipta prostrata* Linne) and has long been used to prevent or treat various liver and kidney diseases [17]. Modern research supports the usefulness of FLL for the treatment of backache, blurred vision, insomnia, menopausal symptoms, palpitations, rheumatic pains, and tinnitus and for alleviating age-related symptoms [18,19]. Furthermore, FLL has been shown to have anti-inflammatory effects in mouse peritoneal macrophages [20], and recently, a phenol glycosides extract of FLL was reported to alleviate depressive-like behavior by inhibiting neuroinflammation in mouse hypothalamus [21]. However, evidence-based medicinal proof of therapeutic efficacy of FLL in neuroinflammation is lacking. 

For decades, numerous chemical compounds have been identified in FLL including triterpenoids, flavonoids, iridoids, phenylethanoid glycosides, etc., and triterpenoids are a main compound type of high content [22,23]. In particular, FLL contains large amounts of representative triterpenoids oleanolic acid and ursolic acid, and oleanolic acid has been used as a quality control standard for FLL [24]. Identification of the components of FLL and their pharmacological studies support that FLL has beneficial effects on various health conditions. Both oleanolic and ursolic acids have been reported to have antiviral activity against hepatitis C virus, and oleanolic acid has shown hepatoprotective and immunomodulatory effects [25]. Furthermore, hydroxytyrosol, salidroside, and several secoiridoid glucosides such as lucidumoside B and C, neonuezhenide, oleoside dimethyl ester, and oleuropein has been reported to have antioxidant activities [25]. 

In the present study, we investigated the effect of an extract of FLL (FLLE) and the mechanism responsible for its inhibitory effects on inflammatory response in LPS-stimulated murine BV2 microglial cells.

## 2. Results

### 2.1. Effects of Fructus Ligustri Lucidi Extract (FLLE) on Inflammatory Response Induced by Lipopolysaccharide (LPS) Stimulation in BV2 Cells

**Figure 1:**
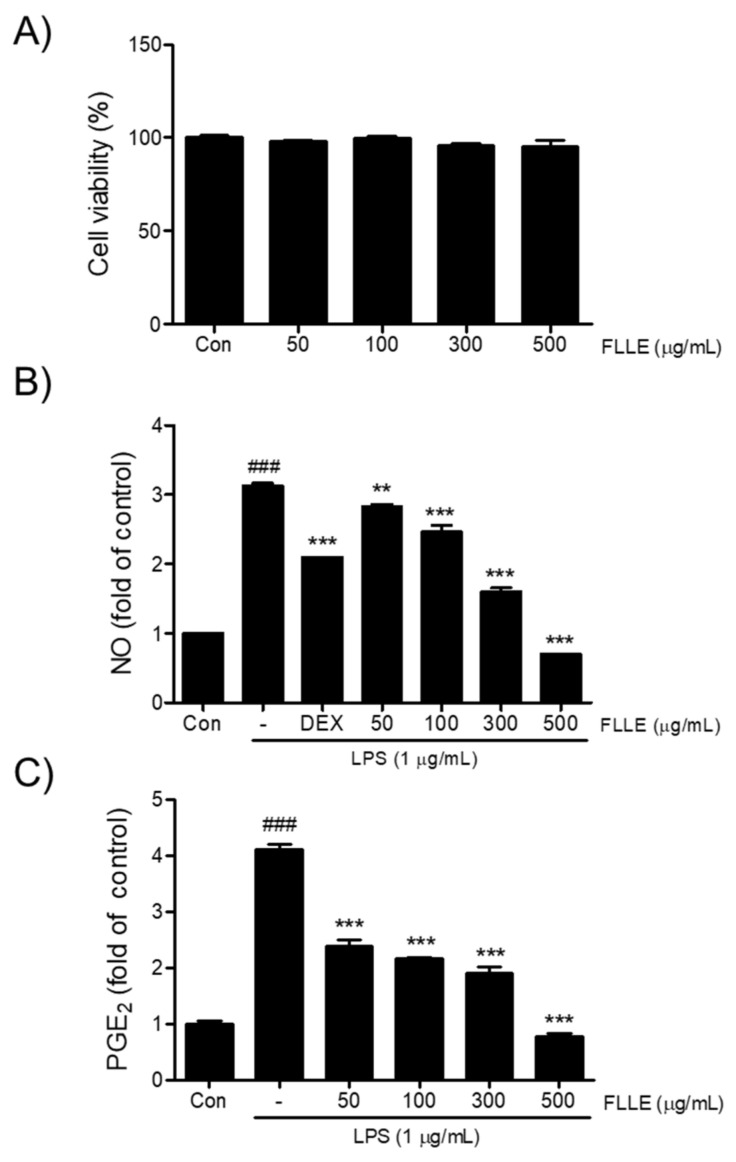
Effects of *Fructus ligustri lucidi* extract (FLLE) on the cell viability of BV2 and on levels of lipopolysaccharide (LPS)-induced inflammatory mediators. (**A**) Cytotoxicity evaluation of FLLE using MTT [3-(4,5-dimethylthiazol-2-yl)-2,5-diphenyl-tetrazolium bromide] assay. BV2 microglia were treated with different concentrations of FLLE (i.e., 50, 100, 300, or 500 μg/mL) for 24 h. Microglia viabilities were determined using an MTT assay. (**B**) Inhibitory effects of FLLE on LPS-induced NO production. (**C**) Inhibitory effects of FLLE on LPS-induced PGE_2_ secretion. Significant vs. vehicle-treated controls, ^###^ *p* < 0.001; significant vs. LPS controls, ** *p* < 0.01, *** *p* < 0.001.

To determine an appropriate concentration for the FLLE treatment of BV2 microglia cells, we first investigated its cytotoxic effect by measuring cell viability. As shown in Figure 1A, more than 95% of cells treated with FLLE at concentrations up to 500 μg/mL for 24 h survived, and thus, FLLE was considered to be non-toxic at concentrations up to this level. Next, we examined the effects of FLLE on the levels of NO and PGE_2_ under inflammatory conditions. Dexamethasone (Dex), a synthetic glucocorticoid with anti-inflammatory and immunosuppressive activity, is known to treat various diseases related to inflammation in the body [26] and was used here as a positive control. LPS stimulation increased NO production in BV2 cells by about 3-fold, whereas FLLE pretreatment suppressed this increase in a dose-dependent manner (Figure 1B). In particular, 300 or 500 μg/mL of FLLE inhibited this increase more than 1 μM dexamethasone (*p* < 0.001). PGE_2_ levels were elevated by about 4-fold versus non-treated controls at 18 h after LPS stimulation (control 442 ± 40.71 pg/mL vs. LPS 1817 ± 69.35 pg/mL, *p* < 0.001) (Figure 1C), but FLLE pretreatment effectively prevented this increase at all concentrations used (*p* < 0.001 vs. LPS).

**Figure 2:**
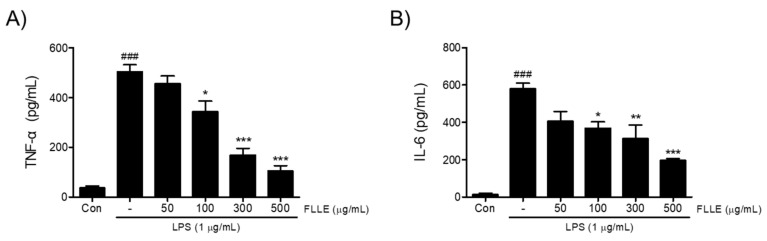
Effects of FLLE on the LPS-induced up-regulations of pro-inflammatory cytokines in BV2 microglia. The up-regulations of tumor necrosis factor-α (TNF-α) (**A**) and interleukin-6 (IL-6) (**B**) by LPS were significantly suppressed by FLLE pretreatment at concentrations from 100 to 500 μg/mL. Significant vs. vehicle-treated controls, ^###^ *p* < 0.001; significant vs. LPS controls, * *p* < 0.05, ** *p* < 0.01, *** *p* < 0.001. Con, vehicle-treated control.

Next, to find out whether FLLE affects the secretions of pro-inflammatory cytokines under neuroinflammatory conditions, we measured amounts of tumor necrosis factor-α (TNF-α) and interleukin-6 (IL-6) secreted into media. TNF-α and IL-6 levels increased by about 14- and 40-fold, respectively, by LPS stimulation (*p* < 0.001, 36.56 ± 13.91 pg/mL vs. 503.67 ± 49 pg/mL for TNF-α and 14.32 ± 11.11 pg/mL vs. 579.39 ± 52.94 pg/mL for IL-6) and these increases were significantly reduced by FLLE pretreatments in the range 100 to 500 μg/mL (Figure 2).

### 2.2. Effects of FLLE on M1 Microglia Polarization by LPS Stimulation in BV2 Cells

**Figure 3:**
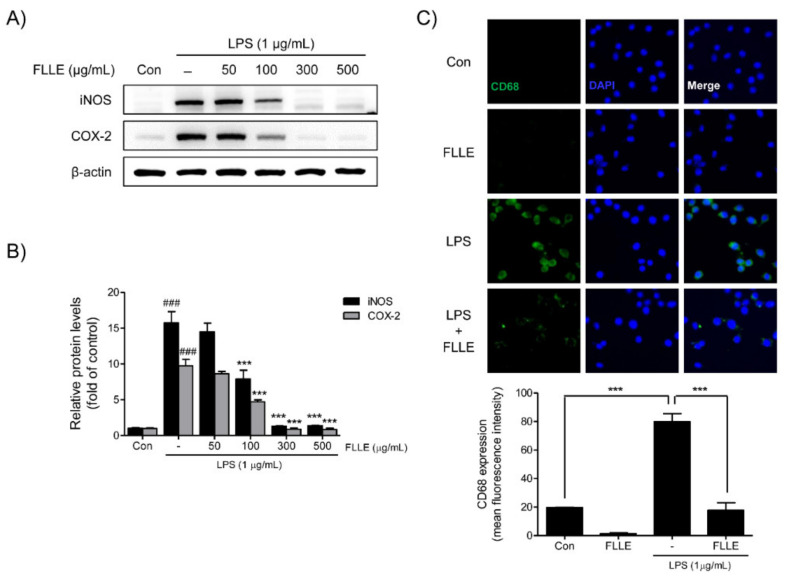
Effects of FLLE on the LPS-induced M1 polarization of BV2 microglia. (**A**) Inhibitory effects of FLLE on LPS-induced inducible nitric oxide synthase (iNOS) and cyclooxygenase-2 (COX-2) levels in BV2 microglia, as determined by western blotting. Representative images are shown and experiments were repeated three times. (**B**) Quantification of iNOS and COX-2 levels relative to β-actin. Results are expressed the means ± standard errors of the means (SEMs) of three independent experiments. Significant vs. vehicle-treated controls, ^###^ *p* < 0.001; significant vs. LPS controls, *** *p* < 0.001 (**C**) Representative immunofluorescent images of CD68 expression in FLLE-treated BV2 cells under normal or inflammatory conditions. CD68: green, DAPI (4,6-diamidino-2-phenylindole): blue.

Western blotting was used to confirm the expressions of inducible nitric oxide synthase (iNOS) and cyclooxygenase-2 (COX-2) in LPS-stimulated BV2 migroclia. iNOS and COX-2 protein levels were markedly augmented after LPS stimulation for 18 h, and FLLE pretreatment at 100 to 500 μg/mL significantly inhibited these increases. In fact, at 300 to 500 μg/mL FLLE prevented these increases (Figure 3A,B). 

We then investigated whether FLLE affects M1 microglia polarization by detecting CD68 (a microglia marker of the active M1 microglial phenotype) by immunofluorescence staining. The results showed numbers of CD68 positive microglia increased after LPS stimulation, and FLLE pretreatment reduced these numbers (Figure 3C), which indicated the attenuation of inflammatory response by FLLE may have been due to the suppression of M1 polarization.

### 2.3. Effects of FLLE on Inflammatory Mitogen-Activated Protein Kinases (MAPK) and Nuclear Factor-Kappa B (NF-κB) Signaling Pathways in BV2 Cells

**Figure 4:**
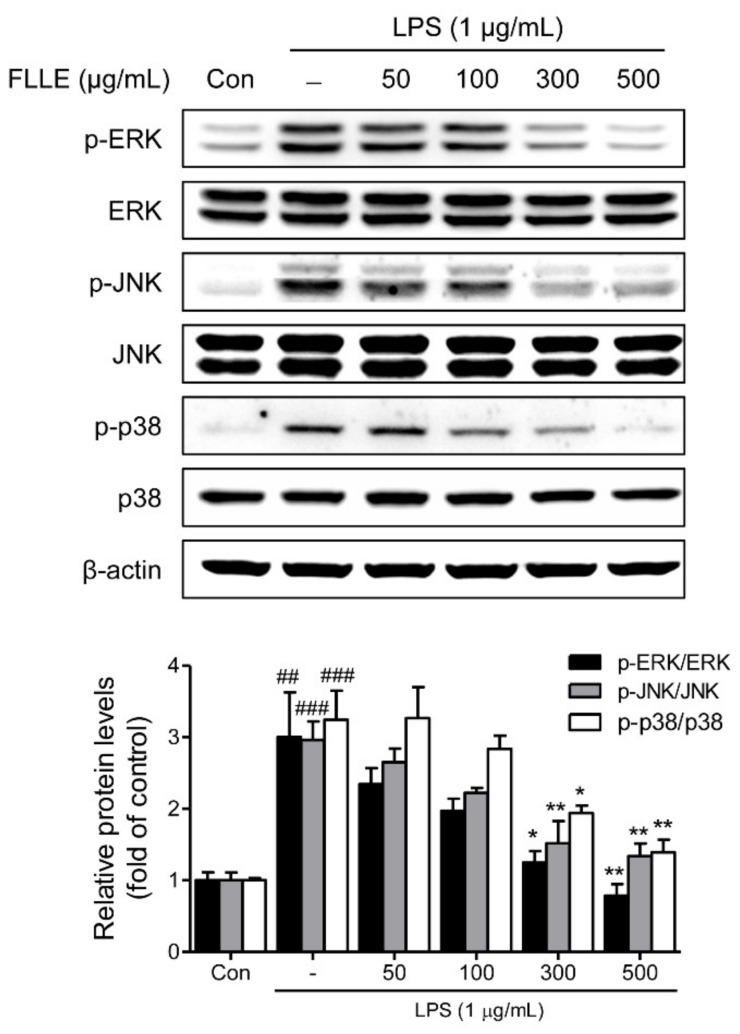
Effects of FLLE on the mitogen-activated protein kinases (MAPK) signaling pathway in LPS-stimulated BV2 microglia. BV2 cells were pre-treated with different concentrations of FLLE for 18 h and then treated with LPS (1 μg/mL) for 30 min. Protein levels of total or phosphorylated MAPKs (i.e., extracellular signal-regulated kinase 1/2 (ERK1/2), c-Jun N-terminal kinase (JNK), and p38 MAPK) were analyzed by western blotting. Representative images are shown and experiments were repeated three times. Significant vs. vehicle-treated controls, ^##^ *p* < 0.01, ^###^ *p* < 0.001; significant vs. LPS controls, * *p* < 0.05, ** *p* < 0.01.

To confirm that FLLE affects inflammatory signaling pathways, we checked the protein levels of MAPK (mitogen-activated protein kinases) subfamilies by western blotting. As was expected, LPS significantly induced phosphorylations of ERK1/2 (extracellular signal-regulated kinase 1/2), JNK (c-Jun N-terminal kinase), and p38 MAPK, and these activations were dose-dependently inhibited by FLLE pretreatment (Figure 4).

**Figure 5:**
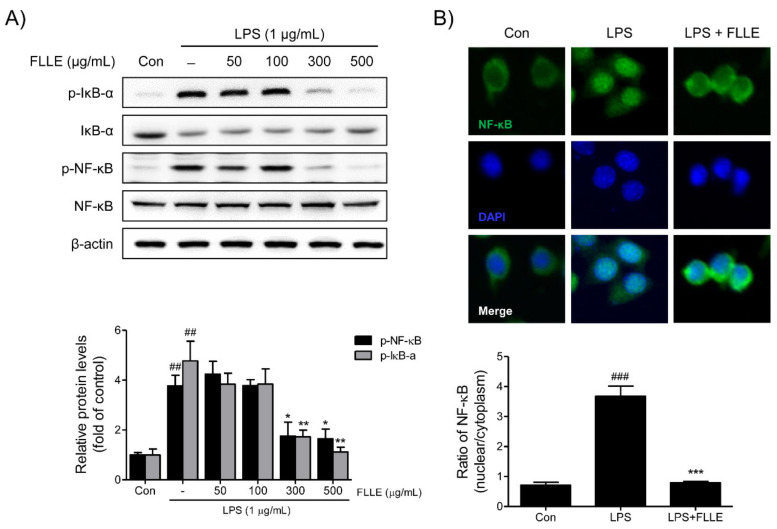
Effects of FLLE on nuclear factor-kappa B (NF-κB) activation in LPS-stimulated BV2 microglia. (**A**) Western blot images of total or phosphorylated NF-κB and IκBα. Representative images are shown and experiments were repeated three times. (**B**) The activation of NF-κB was assessed immunocytochemically. Fluorescence images showed that at 300 μg/mL pretreatment with FLLE inhibited the LPS-induced nuclear translocation of NF-κB in BV2 microglia. p-NF-κB: Green, DAPI: blue. Significant vs. vehicle-treated controls, ^##^ *p* < 0.01, ^###^ *p* < 0.001; significant vs. LPS controls, * *p* < 0.05, ** *p* < 0.01, *** *p* < 0.001.

To determine how FLLE regulates nuclear factor-kappa B (NF-κB) activation under inflammatory conditions, the protein levels of NF-κB and IκBα (regulator of NF-κB) and the nuclear translocation of NF-κB were investigated. FLLE at 300 and 500 μg/mL markedly suppressed the LPS-induced phosphorylations of NF-κB p65 and IκBα (Figure 5A). Furthermore, the LPS-induced nuclear translocation of NF-κB was effectively prevented by 300 μg/mL FLLE as determined by immunofluorescence staining (Figure 5B).

### 2.4. Effects of FLLE on Heme Oxygenase (HO)-1 Expression in BV2 Cells

**Figure 6:**
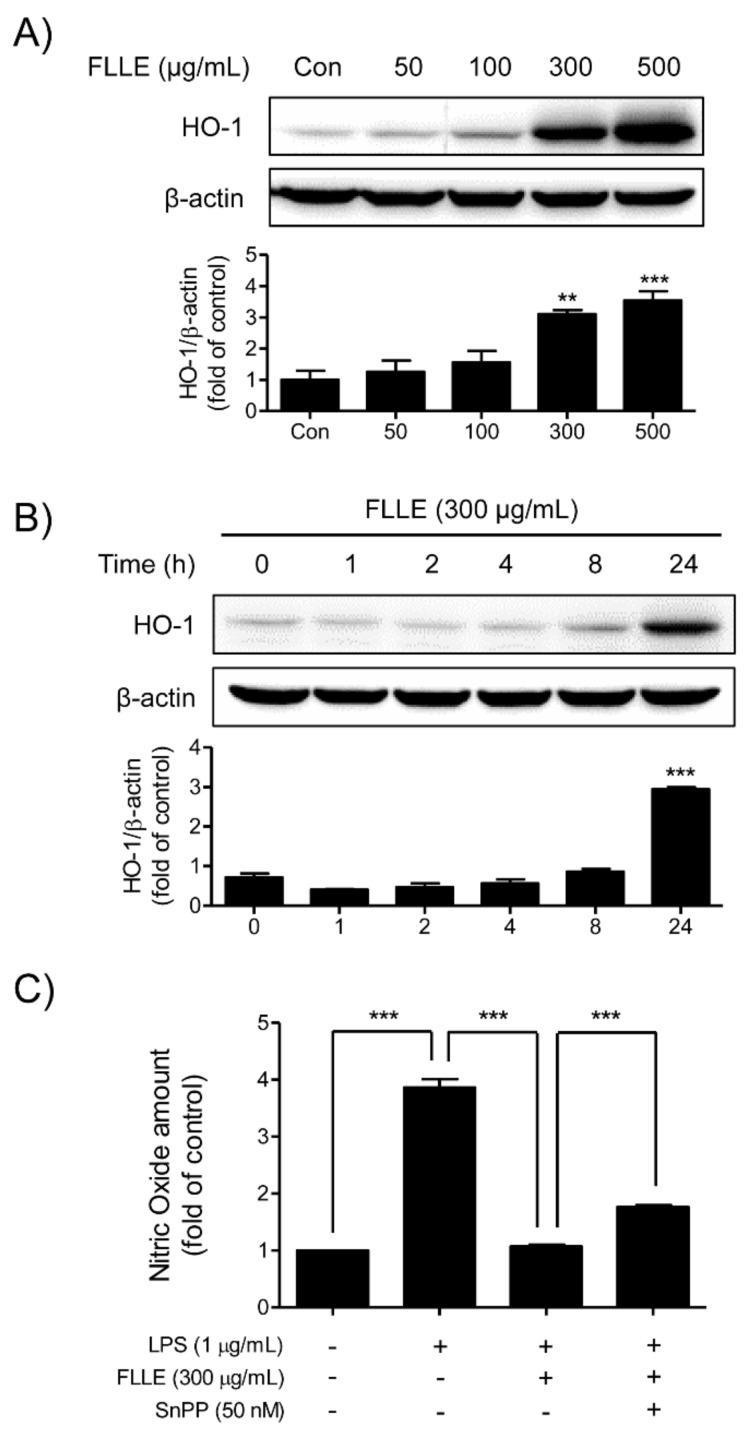
Effects of FLLE on heme oxygenase (HO)-1 expression in BV2 microglia. (**A**) HO-1 protein expression patterns in BV2 cells treated with different concentrations of FLLE. BV2 cells were treated with various concentration of FLLE for 18 h. (**B**) HO-1 protein expression patterns in 300 μg/mL FLLE-treated BV2 microglia at various time points (from 1 to 24 h). Representative images are shown and experiments were repeated three times. (**C**) The role played by HO-1 in the inhibition of NO production by FLLE under inflammatory conditions. Cells were pre-treated with 50 nM SnPP (a HO-1 inhibitor) for 30 min, followed by treatment with 300 μg/mL FLLE for 1 h, and subsequently stimulated with 1 μg/mL LPS for 18 h. NO levels are presented as means ± SEMs. Significant vs. vehicle-treated controls, ** *p* < 0.01, *** *p* < 0.001.

Western blotting and NO assay were used to determine whether FLLE induces the expression of the anti-inflammatory mediator heme oxygenase (HO)-1 in BV2 microglia. FLLE dose-dependently increased levels of HO-1, and the highest expression was observed at 500 μg/mL FLLE (Figure 6A). In addition, at 300 μg/mL FLLE started to induce HO-1 after 8 h of treatment and its expression peaked at 24 h (Figure 6B). These results show FLLE markedly increased HO-1 expression in a time- and dose-dependent manner.

To determine whether FLLE exhibited anti-inflammatory effects by inducing HO-1 expression, we measured amounts of NO in BV2 microglia pretreated with 50 nM Tin protoporphyrin IX (SnPP, a HO-1 inhibitor) for 30 min, then with 300 μg/mL FLLE for 1 h, and finally with 1 μg/mL LPS for 18 h. When BV2 cells were pretreated with SnPP to block HO-1 induction by FLLE, the inhibitory effect of FLLE on the LPS-induced NO production was reduced (Figure 6C). These results indicate that HO-1 induction contributes, at least in part, to the anti-inflammatory effect of FLLE in BV2 microglia.

### 2.5. Effects of FLLE on the Migratory Ability of BV2 Cells

**Figure 7:**
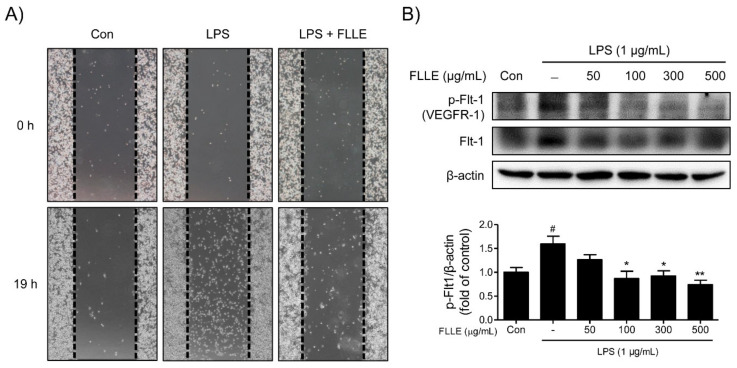
Effects of FLLE on the migration ability of LPS-stimulated BV2 microglia. (**A**) Microscopic images of migration assay results. Photographs were taken of gaps observed under a microscope at different times (0 and 19 h) and dotted lines show starting line before moving. (**B**) Western blot band images of total and phosphorylated vascular endothelial growth factor receptor 1 (VEGFR1) in FLLE pretreated BV2 microglia under inflammatory conditions. Pretreatment with FLLE inhibited LPS-induced VEGFR1 overexpression in BV2 microglia. Representative images are shown and experiments were repeated three times. Significant vs. vehicle-treated controls, ^#^ *p* < 0.05; significant vs. LPS controls, * *p* < 0.05, ** *p* < 0.01.

The effect of FLLE on the migratory ability of microglia was assessed using a wound-healing migration assay. LPS stimulation significantly promoted the migration of BV2 microglia, whereas co-treatment with 300 μg/mL FLLE and LPS had a weaker effect on LPS-induced microglia migration (Figure 7A).

To elucidate the mechanism responsible for the inhibition of BV2 migration by FLLE, we investigated the effects of treatments on the expression pattern of VEGFR1 (vascular endothelial growth factor receptor 1)/Flt-1 (fms-like tyrosine kinase 1) protein. LPS stimulation significantly induced Flt-1 phosphorylation, and FLLE pretreatment dose-dependently reduced the protein levels of phosphorylated VEGFR-1 (Figure 7B). These results indicated the attenuation of BV2 motility by FLLE may have been due to the suppression of VEGFR-1 phosphorylation.

### 2.6. Quality Control of FLLE

**Figure 8:**
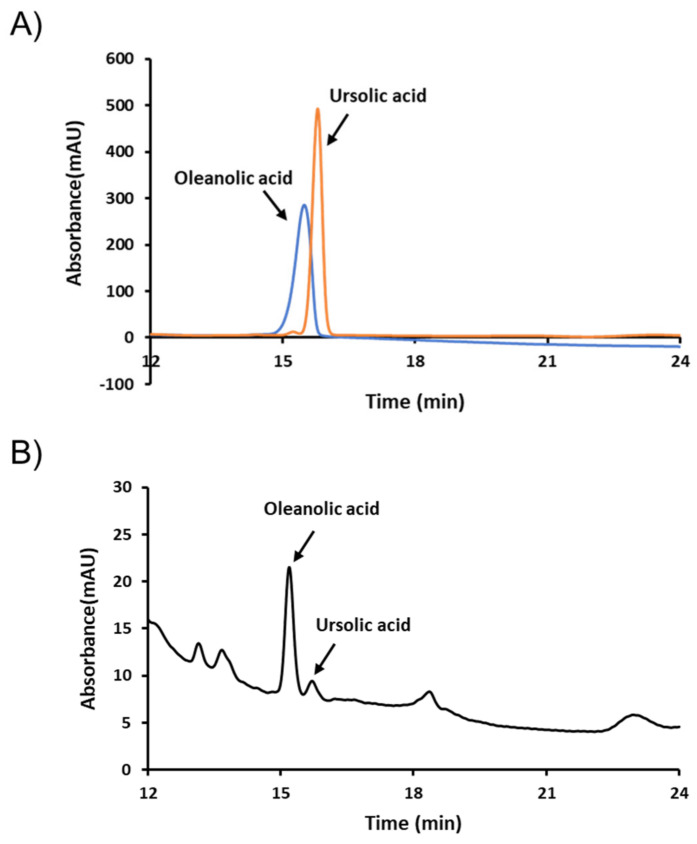
High-performance liquid chromatography (HPLC) chromatograms of FLLE. FLLE and its marker compounds, oleanolic and ursolic acids, were analyzed by HPLC. (**A**) Chromatogram of oleanolic and ursolic acids (**B**) Chromatogram of FLLE.

For quality control (QC) purposes, we selected oleanolic and ursolic acids as QC markers. Typical high-performance liquid chromatography (HPLC) chromatograms of FLLE and its standards oleanolic and ursolic acids are shown in Figure 8. As presented, the retention times of these two components were 15.193 and 15.707 min, respectively. Concentrations of the two compounds in FLLE were determined using calibration curves prepared using oleanolic and ursolic acids standards and were found to be 892.728 and 359.769 mg/kg, respectively.

## 3. Discussion

The remarkable growth of the medical industry witnessed over past decades is more than matched by increasing demands centered on improving quality of life. As we enter what has been described as the era of super-aged societies, disease prevention and health management have become focal points of research efforts. In aging societies, the incidences of brain diseases such as cerebrovascular diseases and neurodegenerative diseases are rapidly increasing and, thus, the prevention, diagnosis, and treatment of these brain diseases have become even more important. To meet these demands, our group continues to try to establish scientific bases that support the use of herbal medicines by identifying medicinal herbs that prevent, improve, or cure neurodegenerative diseases and by elucidating their action mechanisms.

The present study shows that FLLE suppresses LPS-induced neuroinflammation and presents the mechanism involved in murine BV2 microglial cells. For centuries, FLL has been widely used in traditional Chinese medicine to cure hepatitis and aging-related symptoms, and in traditional Korean medicine to detoxify kidneys and liver [27]. Modern pharmacological studies have reported that FLL has antioxidant, anti-inflammatory, anticancer, anti-osteoporosis, and hepatoprotective activities [28,29,30,31,32]. Our results are in line with these findings and show FFL potently inhibits neuroinflammation in LPS-treated BV2 microglial cells.

The pathological processes of neurodegenerative diseases are related to the production of oxidative and inflammatory mediators. Microglia can be activated by infection or brain injury and, when activated, secrete inflammatory cytokines, which have been shown to induce neuron damage and result in neurodegenerative disease [33,34]. The present study demonstrates that FLLE significantly reduces the secretions of proinflammatory cytokines such as TNF-α and IL-6 under inflammatory conditions.

Microglia are categorized into M1 and M2 phenotypes [35]. M1 polarization is associated with pro-inflammatory responses, whereas M2 polarization leads to anti-inflammatory responses [36]. An imbalance of microglial polarization due to excessive M1 activation and M2 dysfunction promotes a neuroinflammatory cascade amplifying neuronal damage [37]. Thus, the regulation of microglia polarization presents a potential target for the treatment of neuroinflammatory disorders [38,39]. In the present study, FLLE effectively inhibited LPS-induced increases in CD68, iNOS, and COX-2 levels in BV2 microglia, and inhibited NO and PGE_2_ levels mediated by iNOS and COX-2. These results suggest that FLLE suppressed M1 polarization by inhibiting LPS-induced markers of M1 activation such as CD68, iNOS, and COX-2.

Activations of MAPK and NF-κB pathways, which are well-known representative inflammatory signaling pathways, result in the secretion of pro-inflammatory cytokines (e.g., TNF-α, IL-1β, IL-6, and IL-8), which trigger inflammatory responses [40,41]. Thus, inhibition of these pathways offers a promising means of blocking or delaying neurodegenerative processes associated with inflammation and microglial activation [42,43]. We confirmed that LPS activated MAPKs, including ERK1/2, JNK, and p38 MAPK, in BV2 cells, and increased the secretions of TNF-α and IL-6. Our results show that FLLE markedly inhibited LPS-induced MAPKs activation in these cells.

NF-κB is a transcription factor of the toll-like receptor 4 signaling pathway, and a key regulator of a variety of inflammatory genes, including iNOS and COX-2, and those of various cytokines and chemokines [44]. Several studies have reported that NF-κB binds to the promoter regions of target genes, and thereby increases the expressions of pro-inflammatory mediators [45,46]. Under normal conditions, NF-κB is restricted to cytoplasm due to complex formation with IκB, but various inflammatory stimuli (e.g., IL-1β, TNF-α, or LPS) result in the phosphorylation and subsequent ubiquitination of IκB protein and the activation of NF-κB [47], which is then free to translocate in the nucleus, where it binds to DNA-binding sites in the promoter regions of many genes, and activates their transcription [48]. Our results show that in BV2 cells, FLLE inhibited the LPS-induced phosphorylations of IκB and the p65 subunit of NF-κB, and that these inhibitions suppressed the nuclear translocation of the p65 subunit of NF-κB.

HO-1, belonging to the family of the heat-shock proteins, is induced in response to oxidative stressors or inflammatory stimuli. HO-1 is known to have anti-oxidative, anti-inflammatory, anti-apoptotic, and immunomodulatory effects [49]. Furthermore, it has been reported that the anti-neuroinflammatory effects of several herbal medicines and phytochemicals, such as Gyejibokryeong-Hwan, *Atractylodis rhizoma Alba*, quercetin, sulforaphane, and tryptanthrin, are due to the induction of HO-1 [50,51,52,53,54]. Similarly, we found that FLLE strongly induced HO-1 activation in BV2 cells and that this inhibited NO production.

In response to pathological stimuli, microglia are activated, migrate to injury sites, and promote inflammatory responses, which lead to chronic neuroinflammation and nerve damage [55]. Microglial motility/migration is known to be regulated by various intracellular signaling pathways [56]. Among these, VEGFR1/Flt-1 (a VEGF receptor subtype) has been reported to play crucial functional roles in mediating microglial chemotactic inflammatory responses [57]. Liu, et al. [58] reported that paeoniflorin, a compound isolated from the root of *Paeonia lactiflora* Pallas, suppressed the overactivation and migration of microglia induced by beta-amyloid by inhibiting the VEGF/Flt-1 axis, which demonstrated that modulating microglial migration can attenuate inflammatory processes in the CNS, and that Flt-1 is a potential target for treating neuroinflammation. In the present study, FLLE attenuated BV2 migration by suppressing the phosphorylation of Flt-1 under inflammatory conditions, which suggests FLLE diminishes neuroinflammation by reducing the migration and activation of microglia.

Finally, we confirmed FLLE contains oleanolic and ursolic acids by HPLC analysis. These naturally occurring pentacyclic triterpene acids are found in many medicinal plants and have been reported to have anti-cancer, anti-hyperlipidemia, and hepatoprotective effects [59,60,61]. In particular, their anti-inflammatory properties have long been known [59]. It has been reported that ursolic acid protects against LPS-induced cognitive impairment by inhibiting the production of pro-inflammatory factors by blocking the p38/NF-κB signaling pathway in mouse brain [62]. Furthermore, Castellano, et al. [63] recently reported that oleanolic acid inhibits LPS-induced BV2 microglial activation. These previous studies and the present study strongly support the notion that oleanolic and ursolic acids contribute to the anti-neuroinflammatory effect of FLLE.

## 4. Materials and Methods

### 4.1. Chemicals and Reagents

PGE_2_ parameter assay kits were obtained from R&D Systems (Minneapolis, MN, USA) and the ELISA (enzyme-linked immunosorbent assay) kits for detection of TNF-α and IL-6 were obtained from Ab Frontier (Seoul, Korea). Primary antibodies for NF-κB, p-NF-κB, IκBα, p-IκBα, JNK, p-JNK, ERK1/2, p-ERK1/2, p38 MAPK, and p-p38 MAPK were purchased from Cell Signaling Technologies (Danvers, MA, USA). Anti-iNOS and anti-p-Flt-1 (Y1213) antibodies were purchased from Novus Biologicals (Littleton, CO, USA) and St John’s Laboratory (London, UK), respectively. Other primary antibodies and HRP (horseradish peroxidase)-linked secondary antibodies were supplied by Santa Cruz Biotechnology (Santa Cruz, CA, USA). SnPP (Tin protoporphyrin IX) was purchased from Porphyrin Products (Logan, UT, USA), and the LPS (*E. coli* endotoxin), 4,6-diamidino-2-phenylindole (DAPI), MTT [3-(4,5-dimethylthiazol-2-yl)-2,5-diphenyl-tetrazolium bromide], Griess reagent, and other reagents were purchased from Sigma-Aldrich (St. Louis, MO, USA).

### 4.2. Preparation of FLLE

*Fructus ligustri lucidi* was supplied by Humanherb (Daegu, Korea), and authenticated by Prof. Sun-Dong Park at the Department of Prescriptions, College of Korean Medicine, Dongguk University, where a voucher specimen was deposited (No. DUMCKM2015-046). Dried fruits of *Ligustrum lucidum* (100 g) were added to 800 mL of 30% ethanol and extracted under reflux at 80 °C for 4 h. The extract was filtered twice using Whatman filter paper (8 μm pore size) and the filtrate was concentrated under reduced pressure at 40 °C using a rotary evaporator (EYELA, Japan). Dry powder (FLLE, 6.4 g) was obtained via lyophilization by using a freeze dryer (EYELA) and stored at –20 °C until required.

### 4.3. High-Performance Liquid Chromatography (HPLC)

FLLE was analyzed using Thermo Scientific^TM^ Ultimate^TM^ 3000 HPLC (Thermo Fisher Scientific, Waltham, MA, USA) to determine oleanolic and ursolic acids (Sigma-Aldrich) contents. Separation was performed using an Inno C18 column (Youngjin Biochrom, Korea, 250 × 4.6 mm, 5 μm) at a column temperature of 30 °C and a flow rate of 1 mL/min over 30 min under isocratic elution mode using 80% acetonitrile as eluant. Detection was performed at 210 nm and data were processed using Chromeleon 6.8 software (Thermo Fisher Scientific).

### 4.4. Cell Culture and Drug Treatment

To investigate the effects of FLLE on microglial cells, we used murine BV2 microglia, which were cultured in DMEM (Dulbecco Modified Eagle Medium; Welgene, Gyeongsan, Korea) supplemented with 1% penicillin-streptomycin (Gibco BRL, Gaithersburg, MD, USA) and 10% heat-inactivated fetal bovine serum (FBS, Welgene) at 37 °C in a 5% CO_2_ incubator (Thermo Fisher Scientific). FLLE was dissolved in culture grade dimethyl sulfoxide (DMSO, AppliChem, Darmstadt, Germany) to prepare a 100 mg/mL stock solution. After 6 h of serum starvation, BV2 microglia were pretreated with FLLE for 1 h and then exposed to 1 μg/mL LPS for 18 h. In another experiment, cells were pretreated with 50 nM SnPP for 30 min, treated with 300 μg/mL of FLLE for 1 h, and then exposed to 1 μg/mL LPS for 18 h.

### 4.5. Cell Viability Assay

BV2 cells were seeded at 1 × 10^4^ cells per well (100 μL) on 96-well plates and incubated overnight. After starvation for 6 h in serum-free media, cells were treated with different concentrations of FLLE (50–500 μg/mL) for 24 h, and then 10 μL of MTT solution (2 mg/mL) were added to each well for another 2 h. After removing supernatants, formazan crystals were fully dissolved in 100 μL of DMSO (Junsei Chemical Co., Tokyo, Japan), and well absorbances were assessed by using a microplate reader (Tecan, Research Triangle Park, NC, USA) at 540 nm.

### 4.6. Nitric Oxide Production Assay

BV2 cells were plated at a density of 1 × 10^6^ cells/mL in 24-well plates. After pre-treatment with various concentrations of FLLE (50, 100, 300, or 500 μg/mL) or 1 μM dexamethasone for 1 h, cells were exposed to 1 μg/mL LPS for 18 h. Supernatants were reacted with an equal volume of Griess reagent in a darkened room for 10 min at room temperature, and the amount of NO was assayed by measuring the absorbance at 540 nm using a microplate reader (Tecan).

### 4.7. Enzyme-Linked Immunosorbent Assay (ELISA)

BV2 cells were pretreated with various concentrations of FLLE for 1 h, stimulated with LPS (1 μg/mL) for 18 h, and supernatants were collected. Amounts of the PGE_2_, IL-6, and TNF-α secreted into media were determined using ELISA kits. Absorbances were measured at 450 nm using a multimode microplate reader.

### 4.8. Cell Migration Assay

BV2 cells were plated at a density of 4 × 10^5^ cells/mL on a 6-well plate and incubated overnight. When the cells were ~90% confluent, wounds were made in confluent monolayers with a sterile 1 mL pipette tip. After washing with phosphate buffered saline (PBS), cells were incubated in DMEM medium supplemented with 5% FBS containing LPS (1 μg/mL) or FLLE (300 μg/mL) plus LPS for 19 h. Phase-contrast photomicrographs were taken in the same fields at 19 h or when wounds had completely healed, whichever occurred first.

### 4.9. Western Blotting

BV2 cells were lysed with radioimmunoprecipitation assay buffer (Thermo Scientific, Rockford, IL, USA) containing protease and phosphatase inhibitor cocktail (GenDEPOT, Barker, TX, USA) and total proteins were quantified using a Pierce^TM^ bicinchoninic acid protein assay kit (Thermo Scientific). Equal amounts of total proteins (40 μg) were subjected to 10% SDS-polyacrylamide gel electrophoresis and transferred onto Amersham^TM^ Hybond^TM^ polyvinylidene fluoride membranes (Sigma-Aldrich). After blocking with 5% skim milk for 1 h at room temperature, membranes were incubated with the primary antibodies at 4 °C overnight. After washing with PBST (phosphate buffered saline with Tween 20), membranes were reacted with HRP-linked secondary antibodies (goat anti-rabbit and goat anti-mouse immunoglobulin Gs (IgGs)) for 1–2 h at room temperature. Blots were developed using Amersham ECL Prime detection reagent (GE Healthcare, Little Chalfont, UK) and images of target protein bands were obtained using a Fusion Solo 2M chemiluminescence imaging system (Vilber Lourmat, Marne-la-vallée, France). Band densities were quantified using ImageJ 1.48v software (NIH Bethesda, Rockville, MD, USA).

### 4.10. Immunofluorescence Microscopy

To investigate the translocation of NF-κB or expression of CD68, BV2 cells were cultivated directly on cover glasses in 6-well plates in the presence of 300 μg/mL FLLE in the absence or presence of LPS (1 μg/mL). Cells were fixed with ice-cold methanol for 10 min, permeabilized in PBS containing 1% Triton X-100 (Sigma-Aldrich) for 10 min, and blocked with 5% bovine serum albumin (GenDEPOT) for 1 h. Cells were then incubated with antibodies for NF-κB or CD68 (1:200) overnight at 4 °C and treated with FITC (fluorescein isothiocyanate)-conjugated goat anti-rabbit (IgG) secondary antibody (1:1000, Invitrogen, Carlsbad, CA, USA) for 1 h at room temperature. Nuclei were counterstained with DAPI for 5 min. After mounting the coverslips on glass slides using ProLong^®^ Gold Antifade Reagent (Thermo Fisher scientific), fluorescence images were captured using a fluorescence microscope (Olympus BX50, Olympus Optical Co., Tokyo, Japan). Fluorescence image analysis was performed using ImageJ 1.48v software (USA). At least three high power fields were selected for analysis of immunofluorescent intensity for CD68 and NF-κB. CD68 expression was measured by analyzing the green fluorescence mean intensity. The fluorescence intensity of NF-κB was determined by dividing the fluorescence intensity in the nucleus by fluorescence intensity in the cytoplasm to detect the ratio of NF-κB translocation from the cytoplasm into the nucleus.

### 4.11. Statistical Analysis

All experiments were performed independently at least three times and results are expressed as means ± standard errors of the means (SEMs). The analysis was performed using one-way analysis of variance (ANOVA) followed by Tukey’s multiple comparisons using Prism 5.0 software (GraphPad Software, Inc., San Diego, CA, USA). Statistical significance was accepted for *p* value < 0.05.

## 5. Conclusions

This study demonstrates the neurobiological activity of FLLE by verifying its inhibitory effect on LPS-induced neuroinflammatory response in BV2 microglial cells. Our results show that FLLE suppresses pro-inflammatory induction in BV2 microglia and suggests that these effects are mediated by its inhibition of the MAPK/NF-κB signaling pathway and the induction of HO-1. Accordingly, our findings suggest that FLL may have the potential to develop as a drug candidate to prevent or relieve neuroinflammation.

## Data Availability

Not applicable.
